# IGF-1 levels in the general population, heart failure patients, and individuals with acromegaly: differences and projections from meta-analyses—a dual perspective

**DOI:** 10.3389/fcvm.2024.1379257

**Published:** 2024-10-31

**Authors:** Yan Hu, Yinling Jiang, Lixia Duan, Songwei Yang, Subinur Tuniyazi, Jianghua Zou, Rui Ma, Gulina Muhemaitibieke, Xiayidanguli Amuti, Yanying Guo

**Affiliations:** Department of Endocrinology, People’s Hospital of Xinjiang Uygur Autonomous Region, Xinjiang Clinical Research Center for Diabetes, Urumqi, China

**Keywords:** insulin-like growth factor 1, heart failure, acromegaly, hormonal, meta-analyses

## Abstract

**Background:**

The complex relationship between insulin-like growth factor 1 (IGF-1) levels and heart failure (HF) is not fully understood, particularly across different populations and conditions. This meta-analysis aims to elucidate the dual perspectives of IGF-1 levels in the general population, HF patients, and individuals with treatment-naïve acromegaly, highlighting IGF-1 as a biomarker and potential therapeutic target in HF management.

**Methods:**

Studies were searched across multiple electronic databases up to January 2024 and independently identified by reviewers. The outcomes were analyzed using RevMan 5.4 and STATA 15.

**Results:**

A total of 25 articles were ultimately included in the analysis. Six studies compared IGF-1 levels between HF patients and non-HF controls, revealing significantly lower IGF-1 levels in HF patients (mean difference −20.93; 95% CI −37.88 to −3.97; *p* = 0.02). This reduction was consistent across various HF subtypes and severities. In addition, individuals with intermediate IGF-1 levels had a lower risk of developing HF [risk ratio (RR) 0.78; 95% CI 0.74–0.83; *p* < 0.01] and HF-related mortality (RR 0.98; 95% CI 0.97, 0.99; *p* < 0.01) compared to those with low IGF-1 levels, suggesting a protective role for maintaining adequate IGF-1 levels. Conversely, treatment-naïve acromegaly patients, characterized by excessively high IGF-1 levels, showed a significantly higher incidence of both diastolic HF [odds ratio (OR) 9.08; 95% CI 6.20–13.29; *p* < 0.01] and systolic HF (OR 13.1; 95% CI 6.64–25.84; *p* < 0.01), implicating supraphysiological IGF-1 levels in adverse cardiac outcomes.

**Conclusions:**

Our meta-analysis highlights the complex interplay between IGF-1 levels and HF. We found that reduced IGF-1 levels are commonly observed in HF patients and are associated with an increased risk of HF and higher HF-related mortality. Conversely, excessively high levels, as observed in acromegaly, are linked to a higher incidence of HF. Based on these results, it is recommended that cardiac function be closely monitored in patients with reduced IGF-1 levels and in those with acromegaly. These findings suggest that IGF-1 could hold potential prognostic value for risk stratification in HF.

## 1 Introduction

Heart failure (HF) stands as a pervasive global health challenge, characterized by high morbidity, elevated mortality rates, poor clinical outcomes, and substantial healthcare costs (1). Depending on the left ventricular ejection fraction (LVEF) measured during the initial echocardiography evaluation, HF is categorized into reduced ejection fraction (HFrEF) and preserved ejection fraction (HFpEF) (2). The New York Heart Association (NYHA) classifies cardiac function into four grades (NYHA I, II, III, and IV) according to the level of activity that precipitates HF symptoms (3). The complexity of HF pathophysiology involves numerous factors, including neurohormonal dysregulation, structural heart changes, and systemic metabolic disturbances (4, 5). One key metabolic regulator that has garnered attention in recent years is insulin-like growth factor 1 (IGF-1), a hormone known for its multifaceted roles in growth, development, and cellular repair processes (6, 7).

IGF-1 is primarily synthesized in the liver in response to growth hormone (GH) stimulation and acts through the IGF-1 receptor, which is expressed in various tissues, including the heart (8, 9). The biological actions of IGF-1 include promoting cell growth, inhibiting apoptosis, and enhancing metabolic efficiency, all of which are crucial for maintaining cardiovascular health (9–11). However, the relationship between circulating IGF-1 levels and HF is complex.

Emerging evidence suggests that IGF-1 plays a pivotal role in cardiovascular health through its anabolic and anti-apoptotic properties (12–14). It enhances cardiac contractility, promotes myocardial cell survival, and facilitates repair mechanisms following injury (10, 15). Animal studies have demonstrated that IGF-1 can improve cardiac function and reduce infarct size in models of myocardial infarction (16). However, the clinical implications of IGF-1 in human heart disease are not as straightforward. Some studies suggest that low IGF-1 levels are associated with worse outcomes in HF (14, 17, 18), while others indicate that high IGF-1 levels, as seen in conditions like acromegaly, might predispose individuals to HF (19, 20). This highlights the need for a nuanced exploration of the role of IGF-1 in HF.

This meta-analysis aimed to provide a comprehensive evaluation of IGF-1 levels in different populations, including the general population, HF patients, and individuals with treatment-naïve acromegaly, a condition characterized by excessive GH secretion and elevated IGF-1 levels. By examining IGF-1 levels in these distinct groups, our study contributes to the existing body of knowledge by offering dual perspectives on how IGF-1 (both deficiency and excess) impacts HF development and progression. This meta-analysis can help identify IGF-1 as a potential biomarker for HF risk stratification and a target for therapeutic interventions, ultimately improving patient outcomes.

## 2 Methods

### 2.1 Search strategy

This meta-analysis aimed to compare IGF-1 levels between HF patients and non-HF controls, across different HF subtypes, and to evaluate the prognostic implications of IGF-1 levels in the general population, HF patients, and treatment-naïve acromegaly patients. Our meta-analysis adhered to the Meta-analyses of Observational Studies in Epidemiology (MOOSE) guidelines (21). We searched for original studies published in English using electronic databases (PubMed, Embase, Web of Science, Cochrane Library, the Web of Science, Scopus, and Google Scholar) from their inception until January 2024. The search strategy combined MeSH terms and free-text words related to IGF-1, HF, and acromegaly. Specific search terms included (insulin-like growth factor I or IGF-1) or (acromegaly or growth hormone-secreting pituitary adenoma) and (heart failure or heart failure, systolic or heart failure, diastolic). The search history for PubMed is presented in Supplementary Table S1.

### 2.2 Inclusion and exclusion criteria

Two authors independently screened all articles according to the following inclusion criteria: (i) and (ii), (i) and (iii), or (i) and (iv). The inclusion criteria included the following: (i) studies with participants older than 18 years using an epidemiologic study design (e.g., case–control, cohort study, or nested case–control); (ii) studies reporting IGF-1 levels between HF patients and non-HF controls, HFrEF and HFpEF, NYHA grade III–IV and NYHA grade I–II, or non-survivors and survivors of HF; (iii) studies providing hazard ratios (HRs), risk ratios (RRs), or odds ratios (ORs) with 95% confidence intervals (CIs) for HF or death from HF based on different IGF-1 levels; and (iv) studies presenting data on the occurrence of diastolic HF, systolic HF, or left ventricular hypertrophy (LVH) in treatment-naïve acromegaly patients compared to healthy controls. If multiple studies were conducted in the same population, the most recent or most relevant study was selected for analysis.

Exclusion criteria included the following: (i) studies lacking sufficient data for synthesis; (ii) review articles, commentaries, case reports, or letters to the editor; and (iii) non-clinical human trials.

### 2.3 Data extraction and quality assessment

Two authors independently selected articles, reviewed full texts, and extracted data from eligible studies. Disputes were resolved by a third independent reviewer. Extracted data included IGF-1 levels in HF patients vs. non-HF controls, HFrEF vs. HFpEF, NYHA grade III–IV vs. NYHA grade I–II, and non-survivors vs. survivors of HF. We also collected HRs, RRs, or ORs with 95% CIs of IGF-1 for HF or death from HF, as well as the number of diastolic HF, systolic HF, or LVH cases in treatment-naïve acromegaly patients compared to healthy controls. Basic study information, including the first author's name, publication year, study country, study design, criteria for HF, number of participants, and mean age, were recorded. The quality of the included studies was assessed by two additional authors according to the Newcastle–Ottawa Scale (NOS) (22), which awards stars across three domains, namely selection, comparability, and exposure or outcome, with total scores indicating low (0–3 stars), medium (4–6 stars), or high (7–9 stars) quality.

### 2.4 Statistical analysis

Meta-analysis and forest plots were applied using RevMan 5.4, while sensitivity analysis was performed using STATA software version 15 (STATA Corp). Continuous variables were presented as standardized mean difference (SMD) or mean difference (MD) with a 95% CI. For dichotomous data, ORs with 95% CIs for each original study were gathered for meta-analysis using the Mantel–Haenszel model. The summary RR with 95% CI was calculated to assess the relationship between circulating IGF-1 levels and the risk of HF or death due to HF, interpreting risk estimates as risk ratios (RRi) (23). We computed the natural logarithms of RRs [log (RR_i_)] with their corresponding standard errors (*s*_i_ = *d*_i_/3.92), where *d_i_* is the log(upper bound 95% CI of RR_i_) − log(lower bound 95% CI of RR_i_). If the original study used the higher IGF-1 category as the comparison group, the effective count method described by Hamling et al. was applied to transform the comparison to the lower IGF-1 category (24). *p* < 0.05 was considered statistically significant.

Heterogeneity was assessed using the chi-square (*X*^2^) test and presented as inconsistency index (*I*^2^) values (25). *I*^2^ < 50% and *p* > 0.1 indicated little heterogeneity among studies, allowing the use of a fixed-effects model. If heterogeneity (*I*^2^ > 50%) was unexplained and within acceptable limits, a random-effects model was used (26). Egger's regression analysis was performed to explore sources of heterogeneity when more than 10 studies were included. Subgroup analyses were conducted to identify the reliability of the meta-analysis, and sensitivity analysis was performed to examine the influence of individual studies on the pooled results. Publication bias was evaluated using funnel plots when more than 10 studies were available (27, 28).

## 3 Results

### 3.1 Study identification

**Figure 1:**
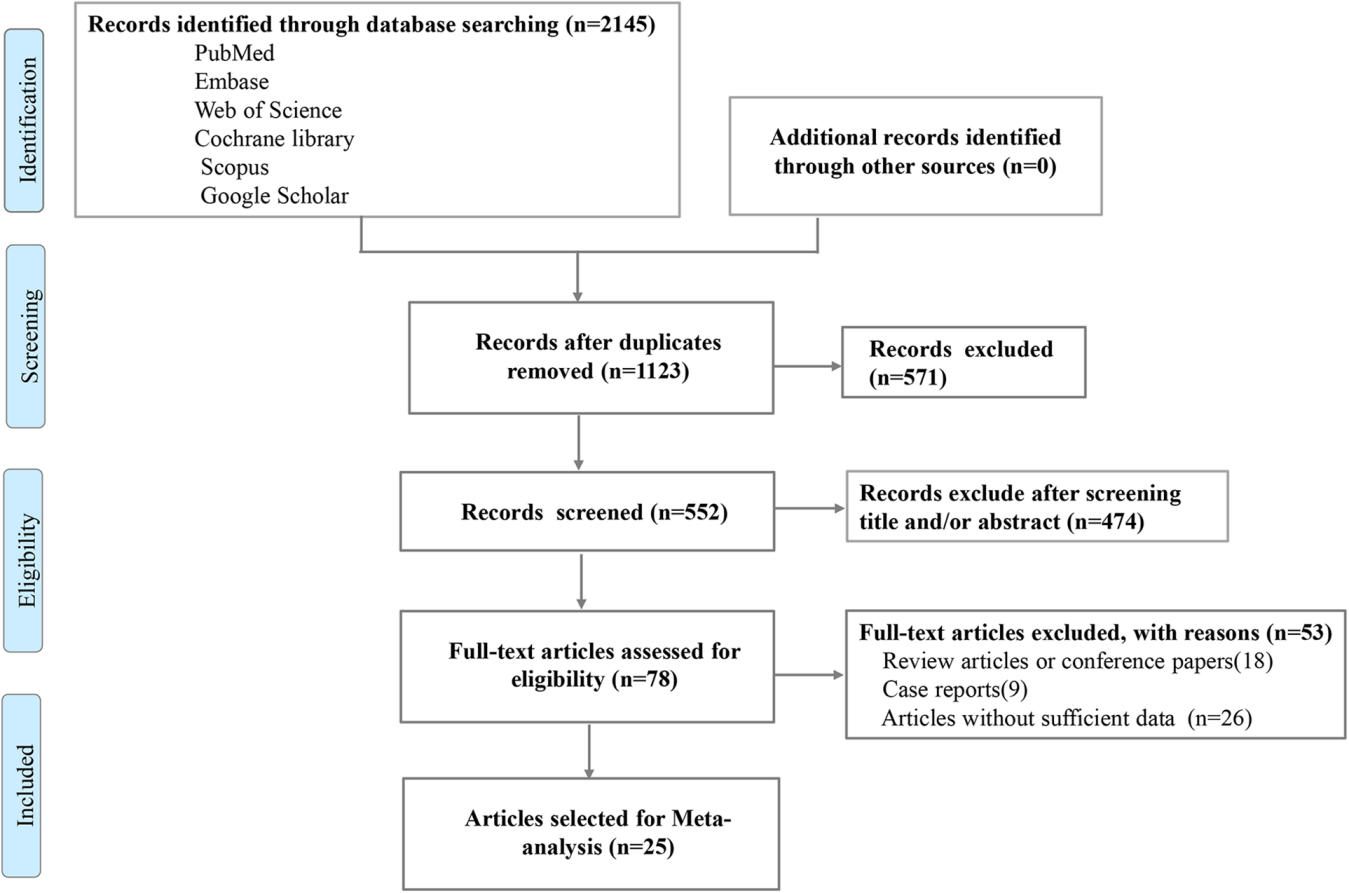
Flowchart showing the selection strategy of our current meta-analysis.

A total of 2,145 articles were retrieved from electronic databases. After removing duplicates, screening titles and abstracts, and conducting a comprehensive study assessment, 25 articles were ultimately included. The search and identification procedures are illustrated in Figure 1.

### 3.2 Detailed information on the selected trials

In this meta-analysis, six studies compared IGF-1 levels between HF patients and non-HF controls (29, 18, 30–33). Three articles reported HRs or ORs for HF in individuals with intermediate IGF-1 vs. low IGF-1 levels (34–36), and five studies reported HRs or RRs for death due to HF in patients with intermediate IGF-1 vs. low IGF-1 levels (29, 37–40). Furthermore, three studies compared IGF-1 levels between HFrEF and HFpEF patients (30, 32, 33), four studies compared IGF-1 levels between HF patients classified as NYHA grade III–IV and those classified as NYHA grade I–II (31, 39–41), and four studies compared IGF-1 levels between non-survivors and survivors of HF (38, 40, 42, 43). Detailed information is provided in Table 1. Seven studies reported cases of diastolic HF in treatment-naïve acromegaly patients and healthy controls (44–50), 4 studies reported cases of systolic HF in treatment-naïve acromegaly patients and healthy controls (47–49, 51), and 10 studies reported cases of LVH in treatment-naïve acromegaly patients and healthy controls (44–53). The characteristics of the included studies involving acromegaly patients and control groups are presented in Table 2. The quality assessment of eligible studies is presented in Supplementary Table S2, with 2 articles scoring 9 points, 5 articles scoring 8 points, 10 articles scoring 7 points, and 8 articles scoring 6 points according to the NOS scoring system. No studies were excluded due to low quality, indicating that all studies are of medium or high quality.

**Table 1:** Main characteristics of the studies included in the meta-analysis of IGF-1 levels and HF.

Reference	Study design	Country	Comparisons	Indicators collected
Andreassen et al. (29)	Prospective cohort	Denmark	HF vs. control	IGF-1
			Intermediate IGF-1 vs. low IGF-1 levels	HR for deaths due to HF
			HF non-survivors vs. HF survivors	IGF-1
Anker et al. (18)	Cross-sectional	Germany	HF vs. control	IGF-1
Barroso et al. (17)	Cross-sectional	Germany	HF vs. control	IGF-1
			HFrEF vs. HFpEF	IGF-1
Broglio et al. (31)	Cross-sectional	Italy	HF vs. control	IGF-1
			NYHA grade III–IV vs. NYHA grade I–II	IGF-1
Faxen et al. (32)	Prospective cohort	Sweden	HF vs. control	IGF-1
			HFrEF vs. HFpEF	IGF-1
Guo et al. (33)	Prospective cohort	China	HF vs. control	IGF-1
			HFrEF vs. HFpEF	IGF-1
Jankowska et al. (39)	Prospective cohort	Poland	NYHA grade III–IV vs. NYHA grade I–II levels	IGF-1
			Intermediate IGF-1 vs. low IGF-1 levels	HR for deaths due to HF
Petrettak et al. (40)	Prospective cohort	Italy	NYHA grade III–IV vs. NYHA grade I–II	IGF-1
			HF non-survivors vs. HF survivors	IGF-1
			Intermediate IGF-1 vs. low IGF-1 levels	HR for deaths due to HF
Watanabe et al. (41)	Retrospective cohort	Japan	NYHA grade III–IV vs. NYHA grade I–II	IGF-1
Eshak et al. (38)	Nested case–control	Japan	HF non-survivors vs. HF survivors	IGF-1
			Intermediate IGF-1 vs. low IGF-1 levels	RR for deaths due to HF
De Giorgi et al. (42)	Prospective cohort	Italy	HF non-survivors vs. HF survivors	IGF-1
Vasan et al. (36)	Prospective cohort	USA	Intermediate IGF-1 vs. low IGF-1 levels	HR for the occurrence of HF
Jörn Schneider et al. (34)	Cross-sectional	Germany	Intermediate IGF-1 vs. low IGF-1 levels	HR for the occurrence of HF
Lin et al. (35)	Prospective cohort	China	Intermediate IGF-1 vs. low IGF-1 levels	HR for the occurrence of HF
Arcopinto et al. (37)	Prospective cohort	Italy	Intermediate IGF-1 vs. low IGF-1 levels	HR for deaths due to HF

IGF-1, insulin-like growth factor I; HF, heart failure; HR, hazards ratio; HFrEF, heart failure with reduced ejection fraction; HFpEF, heart failure with preserved ejection fraction; NYHA, New York Heart Association; RR, risk ratio; OR, odds ratio.

**Table 2:** Main characteristics of the studies included in the meta-analysis of acromegaly patients and control groups.

Reference	Study design	Acromegaly patients			Information for acromegaly			Controls			Diagnostic criteria for acromegaly
		Total *N*	Age (years)	Sex (M)	GH	IGF-1	Disease duration (years)	Total *N*	Age (years)	Sex (M)	
Berg et al. (52)	Case–control	13	54.7	11	NA	5.2 SDS	2.8	65	55.4	55	Serum GH > 1 ng/ml after a 75-g OGTT and elevated serum IGF-1 level, matched for age and gender (>2 SDS)
Bondanelli et al. (45)	Cross-sectional	6	32.16	6	18.58 μg/L	828.5 μg/L	4.16	10	30.5	6	Fasting GH levels above 2.5 or 1 μg/L after a 75-g OGTT, in the presence of elevated IGF-1 levels, adjusted for age and gender
Ciulla et al. (47)	Cross-sectional	10	39	6	22.1 μg/L	128.2 nmol/L	NA	10	38	6	Serum GH level > 2.5 μg/L and not suppressible below 1 μg/L after a 75-g OGTT, with elevated circulating IGF-1 levels (age- and sex-adjusted)
Colao et al. (51)	Cross-sectional	25	31.2	12	43.8 μg/L	772 μg/L	5.3	25	30.6	12	GH not suppressible to less than 2 μg/L after a 75-g OGTT, with elevated plasma IGF-1 levels, adjusted for age
Colao et al. (48)	Case–control	205	47	108	52.1 μg/L	2.5 UIN	9.25	410	46	216	Fasting GH levels above 2.5 or 1 μg/L after a 75-g OGTT, in the presence of elevated IGF-1 levels, adjusted for age and gender
Damjanovic et al. (49)	Cross-sectional	102	49.1	44	17.15 μg/L	882.6 μg/L	9.1	33	44.7	13	Elevated basal levels of GH and IGF-1 and non-suppressibility of GH during an OGTT
Vitale et al. (53)	Cross-sectional	97	45.8	44	101.1 mU/L	656.6 μg/L	11.3	97	46.6	44	High serum GH levels measured over 6 h, which were not suppressible below 3 mU/L after a 75-g OGTT, and high plasma IGF-1 levels, adjusted for sex and age
Cansu et al. (46)	Cross-sectional	53	45	21	2.16 ng/ml	285 ng/ml	6.7	22	47	11	Randomly measured GH level > 1 ng/ml and age- and sex-adjusted IGF-1 levels above the normal range
Akdeniz et al. (44)	Cross-sectional	42	50.3	15		454.7 ng/ml	46	30	51.9	11	High age-adjusted serum IGF-1 levels and lack of suppression of GH to less than 1 ng/ml after a 75-g OGTT
Kırış et al. (50)	Cross-sectional	30	46.4	12	61.6 ng/ml	709.5 ng/ml		30	44.3	17	Typical clinical signs and symptoms, random GH > 1 ng/ml, nadir GH > 0.4 ng/ml after 75 g OGTT, and elevated age-adjusted IGF-1 levels above the normal range

GH, growth hormone; IGF-1, insulin-like growth factor I; OGTT, oral glucose tolerance test; SDS, standard deviation score; ULN, upper limit of normal.

### 3.3 Meta-analysis results

#### 3.3.1 Comparison of IGF-1 levels between HF patients and non-HF controls, as well as among different HF subtypes

**Figure 2:**
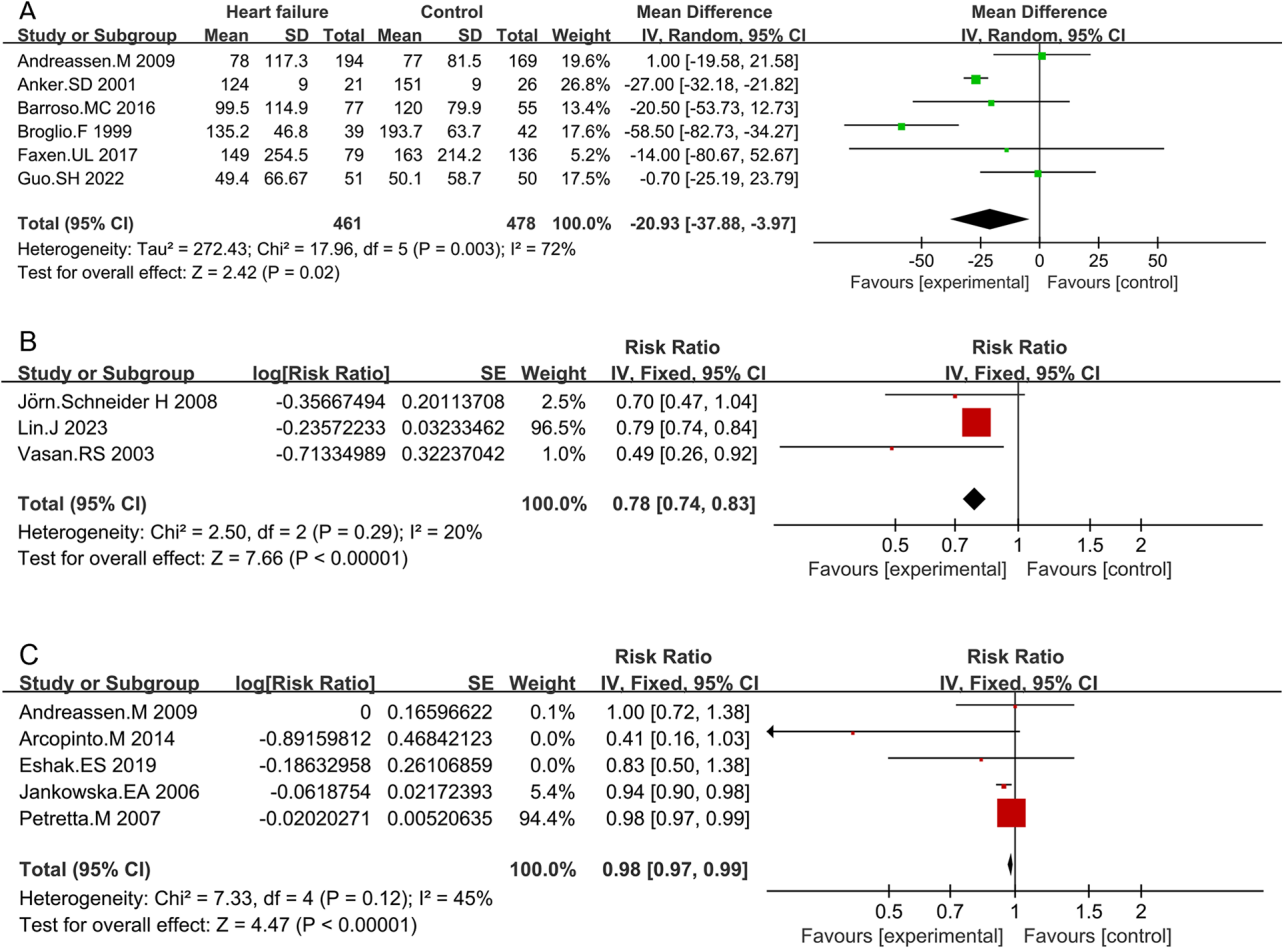
Comparison of IGF-1 levels between HF patients and non-HF controls **(A)**, RR for HF in patients with intermediate IGF-1 vs. low IGF-1 levels **(B)**, and RR for mortality due to HF in patients with intermediate IGF-1 vs. low IGF-1 levels **(C)**.

Six studies compared IGF-1 levels between HF patients and non-HF controls. The characteristics of both HF patients and non-HF controls are detailed in Supplementary Table S4. The circulating IGF-1 level was significantly lower in HF patients than in non-HF controls (MD −20.93; 95% CI −37.88 to −3.97; *p* = 0.02). However, heterogeneity was observed among these studies (*I*^2^ = 72%, *p* = 0.003) (Figure 2 and Supplementary Table S10).

Subgroup analyses based on study design, publication years, and IGF-1 detection methods were conducted (Supplementary Table S3). Cross-sectional studies consistently revealed lower IGF-1 levels in HF patients compared to non-HF controls (MD −34.97; 95% CI −55.98 to −13.95; *p* = 0.001). Studies published before 2010 also indicated lower IGF-1 levels in HF patients (MD −27.34; 95% CI −52.2 to −2.48; *p* = 0.03). Studies utilizing radioimmunoassay methods demonstrated lower IGF-1 levels in HF patients compared to non-HF controls (MD −36.87; 95% CI −62.18 to −11.57; *p* = 0.004). Heterogeneity was not significant in prospective studies (*I*^2^ = 0%, *p* = 0.95), while it was significant in retrospective studies (*I*^2^ = 69%, *p* < 0.01). Higher heterogeneity was observed in studies published before 2010 (85%, *p* = 0.03) compared to those published after 2010 (0%, *p* = 0.40). Studies using radioimmunoassay methods showed significant heterogeneity (69%, *p* < 0.01), whereas those using ELISA did not (0%, *p* = 0.63). Sensitivity analysis indicated that no individual study significantly influenced the pooled MDs, suggesting that the results are robust and credible (Supplementary Figure S1A).

IGF-1 levels were lower in patients with HFrEF compared to those with HFpEF, but the difference was not statistically significant (MD −6.93; 95% CI −25.93 to 12.08; *p* = 0.47) (Supplementary Table S5 and Figure S2). Patients with HF classified as NYHA grade III–IV exhibited lower IGF-1 levels than those classified as NYHA grade I–II (MD −6.66; 95% CI −10.6 to −2.72; *p* < 0.01) (Supplementary Table S6 and Figure S2). IGF-1 levels were significantly lower in HF patients who did not survive compared to those who did (MD −11.68; 95% CI −21.55 to −1.81; *p* = 0.02) (Supplementary Table S7 and Figure S2).

#### 3.3.2 RR for HF in individuals with intermediate IGF-1 vs. low IGF-1 levels

The forest plot combining data from three studies demonstrated a summary RR of 0.78 (95% CI 0.74–0.83; *p* < 0.01) (Supplementary Table S8 and Figure 2). Individuals with intermediate IGF-1 levels showed a lower risk of developing HF compared to those with low IGF-1 levels. No significant heterogeneity was found between studies (*I*^2^ = 20%, *p* < 0.01). Sensitivity analysis indicated that no individual study significantly influenced the pooled RRs, suggesting that the results are robust and credible (Figure S1B).

#### 3.3.3 RR for death due to HF in patients with intermediate IGF-1 vs. low IGF-1 levels

Five studies assessed the RR between IGF-1 concentrations and the risk of death due to HF. Combined data showed that patients with intermediate IGF-1 levels had a lower risk of mortality due to HF compared to those with low IGF-1 levels (RR 0.98; 95% CI 0.97–0.99; *p* < 0.01) (Table S9 and Figure 2). No significant heterogeneity was observed (*I*^2^ = 45%, *p* = 0.12). Sensitivity analysis confirmed that no single study significantly influenced the results (Supplementary Figure S1C).

#### 3.3.4 Risk of developing diastolic HF in patients with treatment-naïve acromegaly

**Figure 3:**
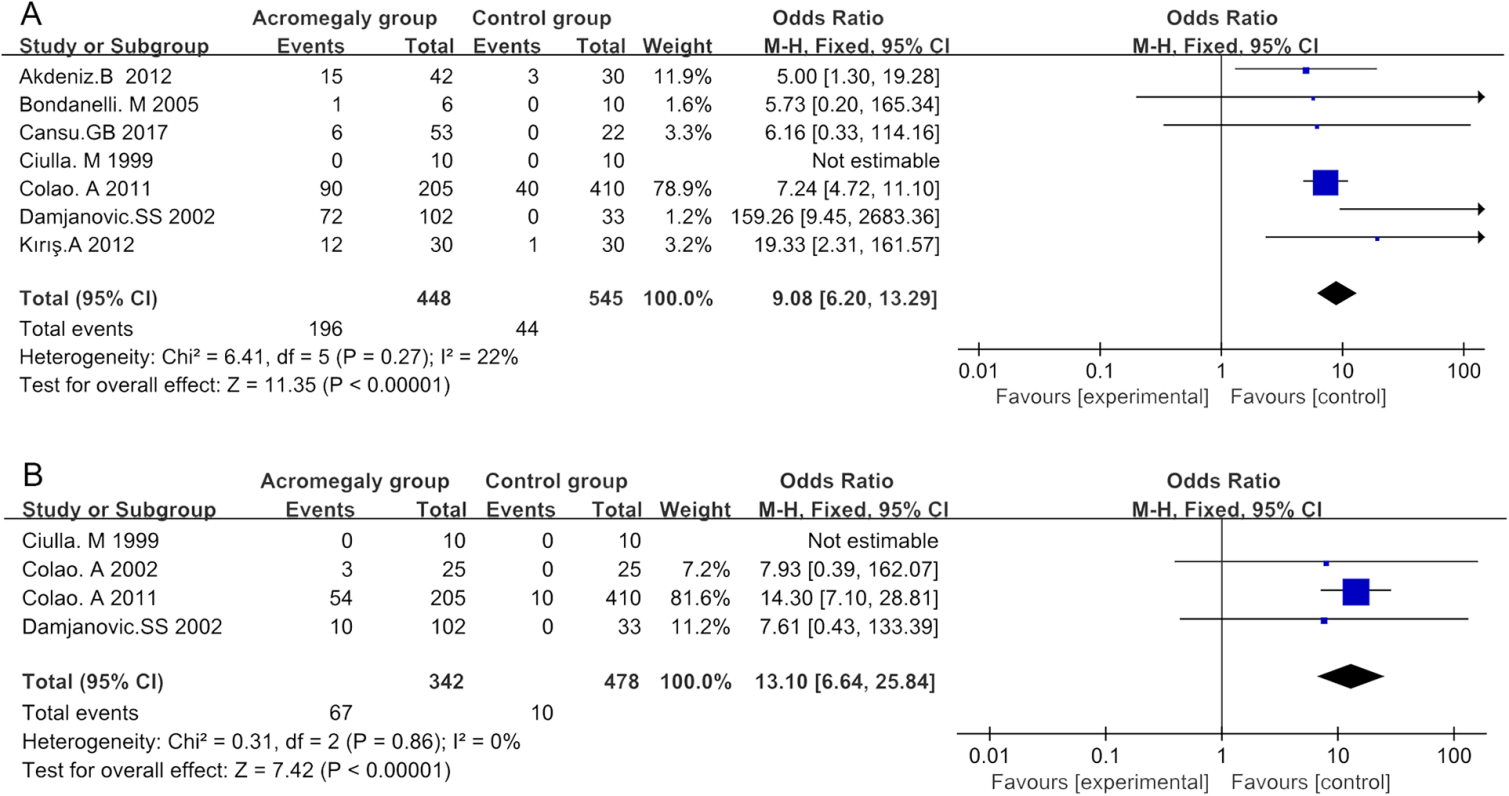
Forest plots depicting the risk of developing HF: diastolic HF **(A)** and systolic HF **(B)** were observed in patients with treatment-naïve acromegaly.

Seven studies, involving a total of 902 individuals, were analyzed. Pooled results demonstrated an overall OR in treatment-naïve acromegaly patients vs. controls (OR 9.08; 95% CI 6.20–13.29; *p* < 0.01) (Table 2 and Figure 3). Patients with treatment-naïve acromegaly had an 8.08-fold increase in the odds of experiencing HF compared to the control group. No significant heterogeneity was found among the studies (*I*^2^ = 22% *p* = 0.27). Sensitivity analysis demonstrated the robustness of these findings (Figure S1D).

#### 3.3.5 Risk of developing systolic HF in treatment-naïve acromegaly patients

Pooled findings from the fixed-effects model indicated a significant difference in OR for HF between treatment-naïve acromegaly patients and the control group (OR 13.1; 95% CI 6.64–25.84; *p* < 0.01) (Table 2 and Figure 3). No significant heterogeneity was detected (*I*^2^ = 0%, *p* = 0.86), and sensitivity analysis confirmed the stability of these results (Supplementary Figure S1E). In addition, pooled findings from the fixed-effects model indicated a significant difference in OR for LVH between treatment-naïve acromegaly patients and the control group (OR 24.65; 95% CI 17.06–35.60; *p* < 0.01) (Table 2 and Supplementary Figure S3). Heterogeneity was minimal (*I*^2^ = 0%, *p* = 0.60), and sensitivity analysis validated the findings (Supplementary Figure S1F).

## 4 Discussion

Our meta-analysis provides a comprehensive evaluation of the complex relationship between IGF-1 levels and HF by examining data from the general population, HF patients, and individuals with treatment-naïve acromegaly. Several intriguing findings warrant further exploration. Patients with HF exhibit lower IGF-1 levels compared to healthy individuals. Reduced IGF-1 levels are associated with an increased risk of HF and higher HF-related mortality, while excessively high levels, as seen in acromegaly, are linked to a higher incidence of HF.

### 4.1 Lower IGF-1 levels in HF patients

The etiologies of HF are complex, with persistent concerns regarding neurohormonal abnormalities (54, 55). A key finding from our meta-analysis is that HF patients exhibit significantly lower IGF-1 levels than non-HF controls. This reduction in IGF-1 levels was consistent across various subgroups, including patients with different HF subtypes (HFrEF vs. HFpEF) and varying degrees of severity (NYHA grade III–IV vs. NYHA grade I–II). These findings suggest an association between the degree of IGF-1 deficiency and the severity of HF. The lower IGF-1 levels observed in HF patients align with previous studies, which suggest that IGF-1 deficiency is associated with adverse cardiovascular outcomes (32, 56). IGF-1 plays a critical role in myocardial function due to its anabolic and anti-apoptotic properties (12, 13, 57). It promotes cardiomyocyte growth, inhibits apoptosis, and enhances metabolic efficiency, all of which are essential for maintaining myocardial integrity and function (9, 58, 59). The reduced IGF-1 levels observed in HF patients may reflect an impaired capacity for myocardial repair and regeneration, contributing to disease progression and increased mortality risk. Our analysis showed that non-survivors of HF had significantly lower IGF-1 levels compared to survivors, underscoring the potential prognostic value of IGF-1 in predicting outcomes for HF patients.

The heterogeneity observed in the studies comparing IGF-1 levels between HF patients and non-HF controls likely stems from variations in factors such as study design, publication year, and IGF-1 detection methods. For instance, cross-sectional studies, older studies, and those utilizing radioimmunoassay methods tended to report lower IGF-1 levels in HF patients. These differences highlight the need for standardized methodologies in future research to enable more consistent comparisons. Differences in the etiology of HF between the original study populations are also a possible source of heterogeneity. Cittadini et al. found that growth hormone deficiency (GHD) may be a major cause of HF in patients with non-ischemic HF (57). Unfortunately, we were not able to obtain data on the etiology of HF in each of the original studies included in this meta-analysis. Follow-up studies could investigate whether there are differences in IGF-1 levels in HF according to etiology.

### 4.2 Protective role of intermediate IGF-1 levels

Another significant aspect of our meta-analysis was examining the relationship between the risk of developing HF and IGF-1 levels. The data indicated that individuals with intermediate IGF-1 levels had a lower risk of developing HF compared to those with low IGF-1 levels. This finding suggests a protective role for maintaining adequate IGF-1 levels in preventing HF onset. IGF-1's beneficial effects on cardiac function, including enhancing contractility, promoting cell survival, and reducing apoptosis, may contribute to this protective effect (60, 61). IGF-1 could serve as a valuable biomarker for stratifying HF patients based on mortality risk, thereby guiding therapeutic decision-making.

### 4.3 IGF-1 levels and mortality in HF patients

Our meta-analysis also explored the relationship between IGF-1 levels and mortality in HF patients. The results showed that patients with intermediate IGF-1 levels had a lower risk of HF-related mortality compared to those with low IGF-1 levels. This finding underscores the potential prognostic value of IGF-1 in HF, suggesting that maintaining or restoring adequate IGF-1 levels could improve survival outcomes.

Physiological levels of IGF-1 play a crucial role in maintaining mitochondrial function and contractility of cardiomyocytes, which are mechanistically linked to HF and its severity (62). First, IGF-1 deficiency impairs mitochondrial function, reducing adenosine triphosphate (ATP) production and increasing reactive oxygen species (ROS), leading to cardiomyocyte apoptosis and contractile dysfunction, which exacerbates HF severity (63, 64). Second, low IGF-1 levels reduce nitric oxide production, increase inflammation, and contribute to endothelial dysfunction, leading to heightened vascular resistance, hypertension, atherosclerosis, and a worsened HF prognosis (65). Third, IGF-1 activates PI3K/Akt pathways, which inhibits apoptosis. IGF-1 deficiency downregulates these pathways, increasing cardiomyocyte apoptosis and loss, further aggravating HF prognosis (8).

### 4.4 IGF-1 and acromegaly: implications for HF risk

Acromegaly, characterized by excessive GH secretion and elevated IGF-1 levels, offers a unique context for examining the effects of high IGF-1 levels on HF risk. Our meta-analysis revealed that treatment-naïve acromegaly patients had a significantly higher incidence of diastolic HF, systolic HF, and LVH compared to healthy controls. International research, including studies involving patients with acromegaly from multiple countries, also demonstrated an increased incidence of HF, which correlated with IGF-1 levels (46, 66). Significantly, overt HF is an indicator of poor prognosis in acromegaly (67), and the condition is linked to higher mortality rates when hormone levels are not effectively managed (68).

These findings highlight the potential detrimental effects of supraphysiological IGF-1 levels on cardiac function. The pathophysiological mechanisms underlying these observations likely involve IGF-1's role in promoting cardiac hypertrophy and fibrosis. Excessive IGF-1 can induce cardiomyocyte hypertrophy and increase wall thickness. This hypertrophy and subsequent fibrosis impair diastolic function, contributing to HF. Excess IGF-1 causes smooth muscle proliferation and arterial stiffness, leading to increased peripheral resistance and exacerbated HF (46).

### 4.5 Clinical implications

The paradoxical effect of IGF-1 underscores the complexity of its role in HF, where both deficiency and excess can lead to adverse outcomes. In line with our research, a comprehensive population-based prospective study demonstrated that individuals with both the lowest and highest levels of circulating IGF-1 had an elevated risk of mortality (69). The clinical implications of our findings are significant: monitoring IGF-1 levels in HF patients could provide valuable prognostic information and help guide therapeutic interventions. For patients with low IGF-1 levels, strategies to restore IGF-1 to intermediate levels might be beneficial. Conversely, managing IGF-1 levels in individuals with acromegaly could be crucial for preventing cardiac complications. This could involve the use of somatostatin analogs or GH receptor antagonists to reduce IGF-1 levels and mitigate the risk of HF.

Despite these insights, our meta-analysis has several limitations. First, heterogeneity in IGF-1 levels was observed between HF patients and non-HF controls, which may limit the generalizability of our results. Second, studies related to acromegaly had small sample sizes, which could be considered a limitation. Despite this, the quality of these studies was high, with low heterogeneity, as determined by the NOS. Given that acromegaly is a rare disease, it is challenging to conduct large-scale studies. Future multi-center studies may help address the issue of limited sample sizes. Third, the limited number of original studies included in this meta-analysis underscores the need for additional research in this area. Future research should focus on longitudinal studies to establish causal links between IGF-1 levels and HF. Efforts to investigate the potential dose–response correlation between IGF-1 levels and HF could provide more comprehensive insights into the role of IGF-1 in HF.

## 5 Conclusion

In summary, this meta-analysis underscores the complex relationship between IGF-1 levels and HF. Reduced IGF-1 levels are commonly observed in HF patients and are associated with an increased risk of HF and higher HF-related mortality. Conversely, excessively high IGF-1 levels, as seen in acromegaly, are linked to a higher incidence of HF. These findings emphasize the importance of maintaining optimal IGF-1 levels for cardiovascular health. It is recommended that echocardiography be routinely conducted for patients with reduced IGF-1 levels and those with acromegaly to assess for any structural and functional cardiac impairments. Identifying IGF-1 as a potential biomarker for HF risk stratification and a target for therapeutic interventions could ultimately improve patient outcomes.

## Data Availability

The original contributions presented in the study are included in the article/Supplementary Material, further inquiries can be directed to the corresponding author.
