# Supplementary material for: IGF-1 levels in the general population, heart failure patients, and individuals with acromegaly: differences and projections from meta-analyses—a dual perspective
Source: Front Cardiovasc Med. 2024 Oct 31;11:1379257. doi: 10.3389/fcvm.2024.1379257 (PMC11560899; doi:10.3389/fcvm.2024.1379257)
Supplement: Supplementary file 1 [file Datasheet1.pdf]

Table S1. The search history on PubMed

Search number	Query	Results
1	Insulin-Like Growth Factor I[Mesh]	36,242
2	(((((((Insulin-Like Growth Factor I[Title/Abstract])) OR (Insulin- Like Somatomedin Peptide I[Title/Abstract])) OR (Insulin Like Somatomedin Peptide I[Title/Abstract])) OR (Somatomedin C[Title/Abstract])) OR (IGF- I-SmC[Title/Abstract])) OR (IGF-1[Title/Abstract])) OR (Insulin Like Growth Factor I[Title/Abstract])	34,734
3	(Insulin-Like Growth Factor I[Mesh]) OR ((((((((Insulin-Like Growth Factor I[Title/Abstract])) OR (Insulin- Like Somatomedin Peptide I[Title/Abstract])) OR (Insulin Like Somatomedin Peptide I[Title/Abstract])) OR (Somatomedin C[Title/Abstract])) OR (IGF- I-SmC[Title/Abstract])) OR (IGF-1[Title/Abstract])) OR (Insulin Like Growth Factor I[Title/Abstract]))	49,300
4	Heart Failure, Diastolic [Mesh] OR Heart Failure, Systolic [Mesh] OR Heart Failure [Mesh]	148,969
5	((Insulin-Like Growth Factor I[Mesh]) OR ((((((((Insulin-Like Growth Factor I[Title/Abstract])) OR (Insulin- Like Somatomedin Peptide I[Title/Abstract])) OR (Insulin Like Somatomedin Peptide I[Title/Abstract])) OR (Somatomedin C[Title/Abstract])) OR (IGF- I-SmC[Title/Abstract])) OR (IGF-1[Title/Abstract])) OR (Insulin Like Growth Factor I[Title/Abstract]))) AND (Heart Failure, Diastolic[Mesh] OR Heart Failure, Systolic[Mesh] OR Heart Failure[Mesh])	223
6	(Acromegaly [Mesh]) OR Growth Hormone-Secreting Pituitary Adenoma [Mesh]	9,650
7	((Acromegaly [Mesh]) OR Growth Hormone-Secreting Pituitary Adenoma [Mesh]) AND (Heart Failure, Diastolic [Mesh] OR Heart Failure, Systolic [Mesh] OR Heart Failure [Mesh])	86
8	(((Acromegaly[Mesh]) OR Growth Hormone-Secreting Pituitary Adenoma[Mesh]) AND (Heart Failure, Diastolic[Mesh] OR Heart Failure, Systolic[Mesh] OR Heart Failure[Mesh])) OR (((Insulin-Like Growth Factor I[Mesh]) OR ((((((((Insulin-Like Growth Factor I[Title/Abstract])) OR (Insulin- Like Somatomedin Peptide I[Title/Abstract])) OR (Insulin Like Somatomedin Peptide I[Title/Abstract])) OR (Somatomedin C[Title/Abstract])) OR (IGF- I-SmC[Title/Abstract])) OR (IGF-1[Title/Abstract])) OR (Insulin Like Growth Factor I[Title/Abstract]))) AND (Heart Failure, Diastolic[Mesh] OR Heart Failure, Systolic[Mesh] OR Heart Failure[Mesh]))	290

Table S2. Quality assessment of included studies

Study	Selection	Comparability	Exposure/outcome	Total score
Andreassen. M 2009	***	*	***	7
Anker.SD 2001	***	*	***	7
Barroso.MC 2016	***	*	**	6
Broglio. F 1999	**	**	***	7
Faxen. UL 2017	***	*	***	7
Guo.SH 2022	***	*	**	6
Jankowska. EA 2006	****	**	***	9
Petretta. M 2007	****	*	**	7
Watanabe. S 2010	**	*	***	6
Eshak.ES 2019	***	**	***	8
De. Giorgi A 2022	****	*	***	9
Vasan.RS 2003	****	*	***	8
Jörn. Schneider H 2008	****	*	***	8
Lin. J 2023	****	*	**	7
Arcopinto. M 2014	***	*	***	7
Berg. C 2013	**	*	***	6
Bondanelli. M 2005	**	**	**	6
Ciulla. M 1999	**	**	**	6
Colao. A 2002	**	*	***	6
Colao. A 2011	***	**	***	8
Damjanovic. SS 2002	***	**	***	7
Vitale. G 2004	***	**	**	7
Cansu. GB 2017	***	**	***	8
Akdeniz. B 2012	**	**	***	7
Kırış. A 2012	**	*	***	6

Table S3 Subgroup analysis of heterogeneity in IGF-1 levels among patients with HF and non-HF controls

Item	Subgroup	No of studies	MD	95%CI	P value	Heterogeneity (I^2)
Study design	Prospective cohort	3	-0.46	-15.79 to 14.87	0.95	0%
	Cross-sectional	3	-34.97	-55.98 to -13.95	0.001	69%
Years of publication	Published before 2010	3	-27.34	-52.2 to -2.48	0.03	85%
	Published after 2010	3	-8.18	-27.08 to 10.73	0.4	0%
IGF-1 detection Methods	radioimmunoassay	3	-36.87	-62.18 to -11.57	0.004	69%
	ELISA	3	-3.53	-17.75 to 10.72	0.63	0%

Abbreviations: IGF-1, insulin-like growth factor I; HF, heart failure; MD, mean difference; CI, confidence interval; ELISA, enzyme-linked immunosorbent assay.

Table S4. Characteristics of HF patients and non-HF controls

Author (year)	Study design	HF				Control			
		Study population	Total N	Age (year)	Sex (M)	Control population	Total N	Age (year)	Sex (M)
Andreassen. M 2009	Prospective cohort study	Patients had a 6-month history of CHF with LVEF < 45%	194	69.3	139	Healthy control subjects with LVEF above 60%, matched for age and gender	169	67.2	119
Anker.SD 2001	Cross-sectional study	Patients with 6-month history of CHF and LVEF < 45%	21			Healthy control subjects	26		
Barroso.MC 2016	Cross-sectional study	HF classified as NYHA II or III, with or without clinical symptoms or signs	79	64	41	Control group with normal cardiac function	55	54	30
Broglio. F 1999	Cross-sectional study	Patients had a history of congestive HF with an LVEF below 40%	39	55.3	36	Healthy control subjects matched for age and gender	42	56	38
Faxen. UL 2017	Prospective, observational, multicenter study	Patients with HF, and LVEF was lower than 40%	79	64	66	Control group with normal cardiac function	136	58	68
Guo.SH 2022	Prospective cohort study	Patients with HF, and LVEF was lower than 40%	151	71	90	The control group included patients with uncontrolled hypertension but normal cardiac function	50	66	21

Abbreviations: HF, heart failure; CHF, chronic heart failure; LVEF, left ventricular ejection fraction.

Table S5. Characteristics of the HFrEF and HFpEF groups

Author (year)	Study design	HFrEF				HFpEF			
		Population	Total N	Age (year)	Sex (M)	Population	Total N	Age (year)	Sex (M)
Barroso.MC 2016	cross-sectional study	NYHA II or III heart failure with or without clinical symptoms or signs	77	73	94	Grade I diastolic dysfunction without clinical HF symptoms of HF	168	66	31
Faxen. UL 2017	prospective, observational, binational, multicenter study	Patients with HFrEF, defined as LVEF < 40%	79	64	41	patients presenting to the hospital with signs and symptoms of acute HF, NTproBNP > 300 ng/L and left ventricular ejection fraction above 45%	85	73	66
Guo.SH 2022	prospective cohort study	Patients with HFrEF, defined as LVEF < 40%	51	68.4	41	Patients with HFpEF defined as LVEF above 50%	70	70.3	30

Abbreviations: HFrEF, heart failure with reduced ejection fraction; HFpEF, heart failure with preserved ejection fraction; NYHA, New York Heart Association; LVEF, left ventricular ejection fraction; HF, Heart failure; NT proBNP, N-terminal pro-brain natriuretic peptide.

Table S6. Characteristics of the NYHA III-IV and NYHA I-II HF groups

Author (year)	Study design	NYHA III-IV			NYHA I-II		
		Population	Total N	Sex(M)	Population	Total N	Sex(M)

Broglio. F 1999	cross-sectional study	Class III and IV heart failure according to the NYHA functional classification	18	94	Class I and II heart failure according to the NYHA functional classification	21	NA
Jankowska. E 2006	prospective cohort study	Class III and IV heart failure according to the NYHA functional classification	73	73	Class I and II heart failure according to the NYHA functional classification	132	132
Petretta. M 2007	prospective cohort study	Class III and IV heart failure according to the NYHA functional classification	57	41	Class I and II heart failure according to the NYHA functional classification	25	NA
Watanabe. S 2010	retrospective cohort	Class III and IV heart failure according to the NYHA functional classification	NA	NA	Class I and II heart failure according to the NYHA functional classification	NA	NA

Abbreviations: NYHA, New York Heart Association; NA, not available.

Table S7. Characteristics between non-survivors and survivors of HF

Author (year)	Study design	Non-survivors of HF			Survivors of HF			Definition of heart failure
		Total N	Age(year)	Sex(M)	Total N	Age(year)	Sex(M)	
Petretta. M 2007	prospective cohort study	17	63		65	63		Patients with clinical signs and symptoms of HF due to idiopathic dilated cardiomyopathy or ischemic heart disease, with a left ventricular ejection fraction <40%
Eshak.ES 2019	nested case-control study	88	69.2	44	88	67.9	44	Patients with decompensated HF

De. Giorgi A 2022	prospective, observational, multicenter study	41	69	35	296	63	237	Patients had CHF with reduced LVEF (<45%) and stable home therapy for at least 3 months
Bhandari. SS 2016	prospective cohort study	537	76.5	352	215			Patients with decompensated HF

Abbreviations: HF, Heart failure; LVEF, left ventricular ejection fraction; CHF, chronic heart failure.

Table S8. Characteristics of studies assessing the RR of IGF-1 in HF

Author (year)	Study design	Population	Follow up	Gender	Adjusted factors	Definition of HF
Jörn. Schneider H 2008	Cross-sectional	55 518 unselected consecutive patients	No follow-up data	Mix	Age, gender, AST, GFR, BMI, diabetes, hypertension, dyslipidemia, and smoking status	Patients with a physician's diagnosis of HF, further supported by laboratory or clinical findings
Lin. J 2023	Prospective cohort study	A total of 394082 participants without CVD and cancers at baseline from UK Biobank	Median 11.6 years	Mix	Age, sex, and BMI.	Congestive HF was diagnosed on the basis of the previously detailed Framingham Heart Study criteria

Vasan.RS 2003	Prospective cohort study	717 elderly individuals who did not have myocardial infarction and congestive HF at baseline.	Mean 5.2 years	Mix	Age, sex, diabetes, systolic blood pressure, hypertension treatment, smoking status, body mass index, total cholesterol–high-density lipoprotein cholesterol ratio, valve disease, prevalent atrial fibrillation, left ventricular hypertrophy on electrocardiography, and prevalent cardiovascular disease	HF is defined and coded as I50 according to ICD-10

Abbreviations: HR, hazard ratio; HF, Heart failure; CVD, cardiovascular disease; BMI, body mass index; ICD-10, the International Classification of Diseases, 10th edition.

Table S9. Characteristics of studies assessing the RR of IGF-1 for non-survivors in HF

Author (year)	Study design	Population	Follow up	Gender	Adjusted factors	Definition of HF
Andreassen. M 2009	Prospective cohort	A total of 194 consecutive HF patients	Median 30 months	Mix	Age	HF is defined and coded as I50 according to ICD-10
Arcopinto. M 2014	Prospective cohort	Population of 207 patients with consecutive CHF, and NYHA classes I-III	Median 3.5 months	Mix	Age, sex, BMI, diabetes, classification according to the NYHA, CKD stage, and NT-proBNP	Patients with CHF in NYHA classes I-III and LVEF of 40% or below
Eshak.ES 2019	Nested case-control	37,769 individuals between the ages of 40 and 79 without a	Median 3.5 years	Mix	Age, sex, and community	HF is defined and coded as I50 according to ICD-10

		history of cardiovascular disease				
Jankowska. EA 2006	Prospective cohort	208 men with CHF, and 366 healthy men	Median 1144 days	Male	A single-predictor model	A history of CHF for more than 6 months, and LVEF less than 45%
Petretta. M 2007	Prospective cohort	82 non-cachectic patients, mean age 61 ± 13 years, with LVEF< 40% and NYHA classes II-IV	Mean 18.4 months	Mix	A single-predictor model	Patients with LVEF< 40% and NYHA classes II-IV.

Abbreviations: HR, hazard ratio; HF, heart failure; ICD-10, the International Classification of Diseases, 10th edition; CHF, chronic heart failure; NYHA, New York Heart Association; CKD, chronic kidney disease; LVEF, left ventricular ejection fraction.

Table S10. Comparison of IGF-1 levels in HF patients and non-HF controls, and among different subtypes of HF

Comparison	Number of studies	Number of cases	Heterogeneity test		Meta-analysis results		
			I2	P Value	Mean Difference (MD)	95% Confidence Interval (CI)	P-Value
HF VS Health controls	6	461/478	72%	<0.01	-20.93	-37.88 to -3.97	0.02
HFrEF VS HFpEF	3	207/323	0%	0.77	-6.93	-25.93 to 22.16	0.47
NYHA III-IV VS NYHA I-II	4	221/250	2%	0.38	-6.66	-10.60 to -2.72	<0.01
Non-survivors VS Survivors	4	190/596	35%	0.21	-11.68	-21.55 to -1.81	0.02

Abbreviations: IGF-1, insulin-like growth factor I; HF, heart failure; MD, mean difference; CI, confidence interval; NYHA, New York Heart Association; HFrEF, heart failure with reduced ejection fraction; HFpEF, heart failure with preserved ejection fraction.

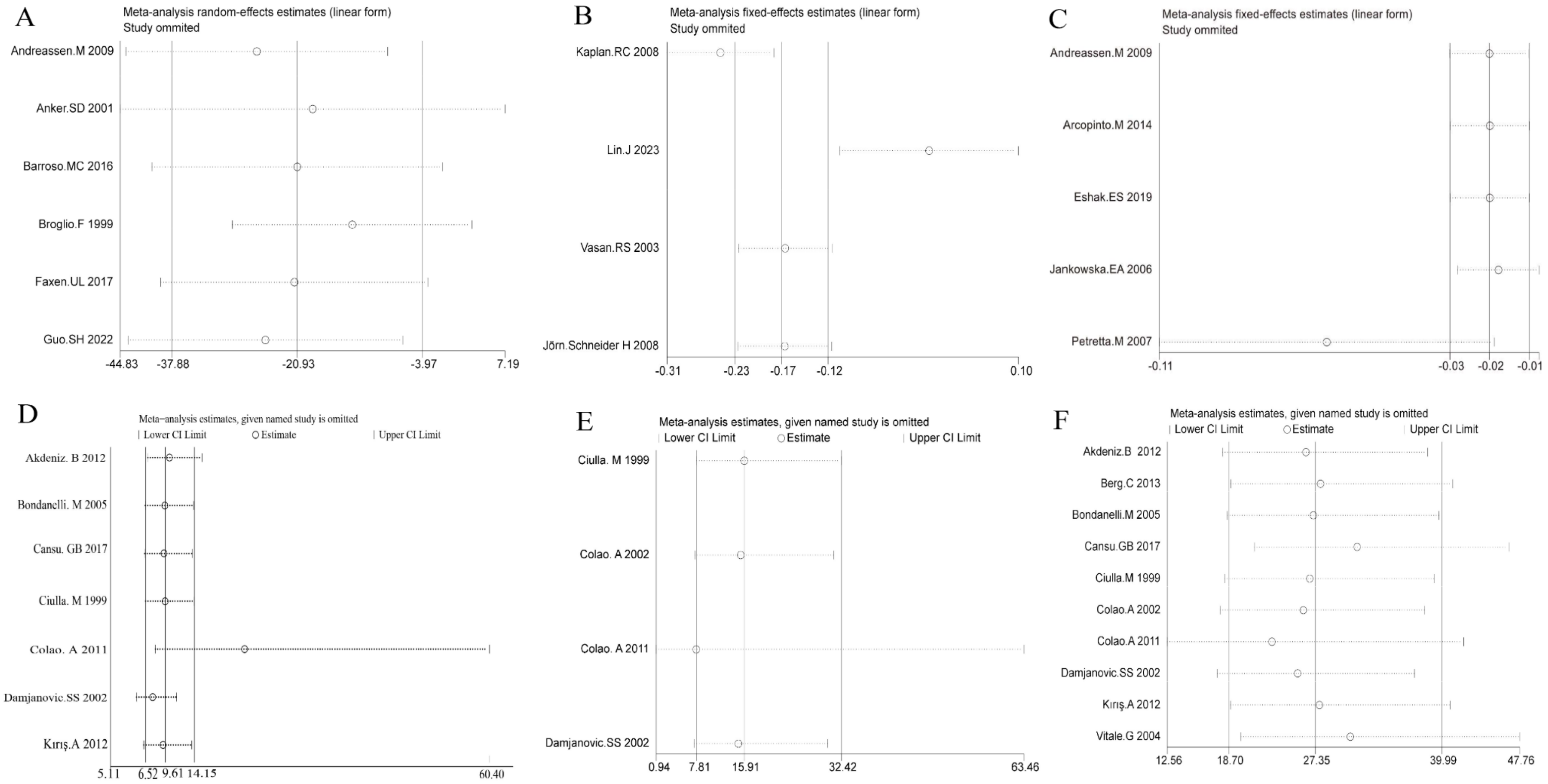

Figure S1. Sensitivity analysis of IGF-1 levels in HF and non-HF controls(A); HF in patients with relatively high and relatively low IGF-1 levels(B); mortality due to HF in patients with relatively high and relatively low IGF-1 levels(C); the risk of developing diastolic HF(D), systolic HF(E), and left ventricular hypertrophy(F) in patients with treatment-naïve acromegaly.

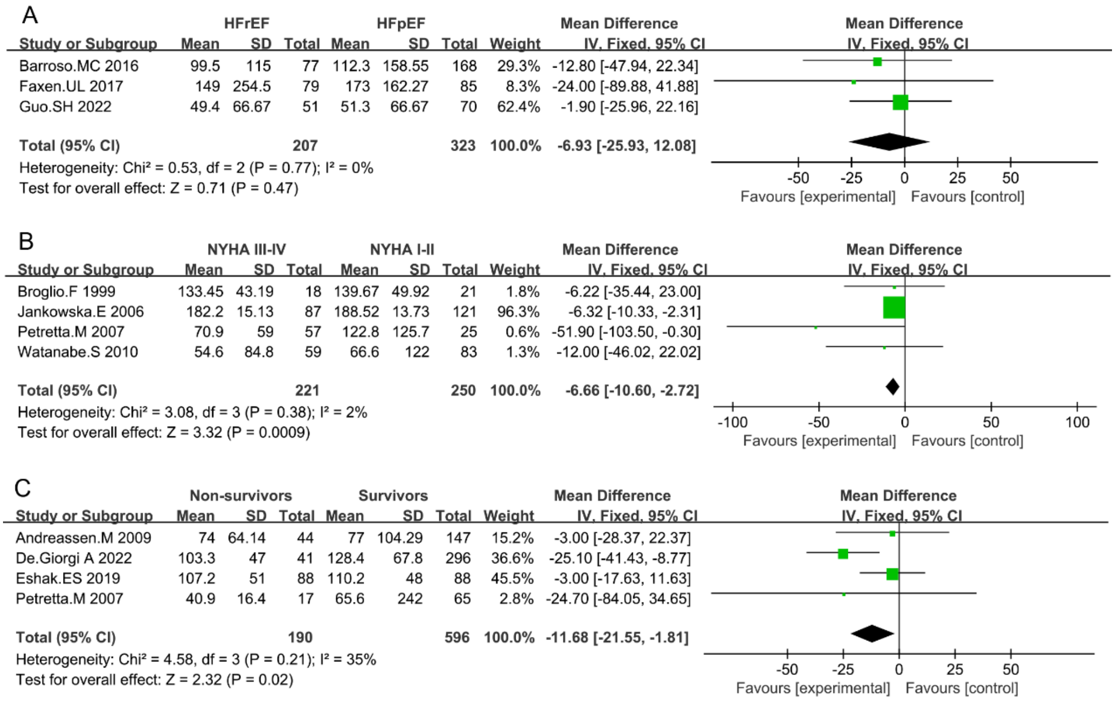

Figure S2. Comparison of IGF-1 levels in HFrEF and HFpEF patients(A); Comparison of IGF-1 levels in NYHA III-IV HF patients and NYHA I-II HF patients (B); Comparison of IGF-1 levels in non-survivors and survivors of HF(C)

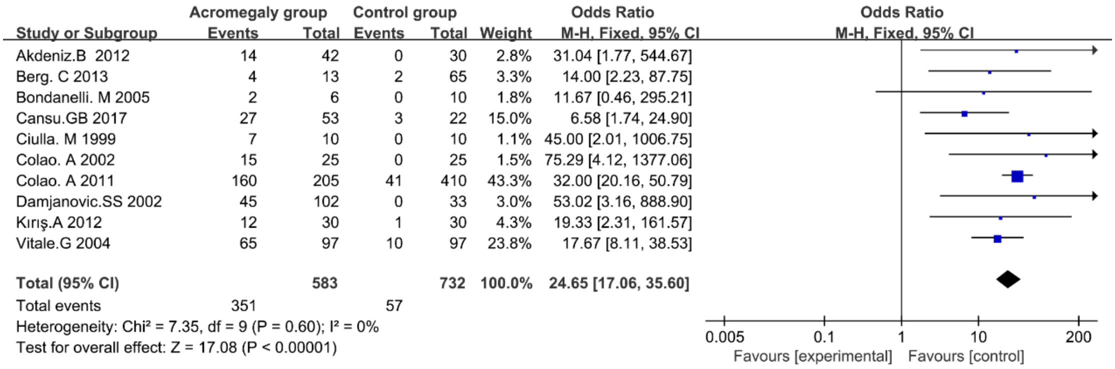

Figure S3. Forest plots depicting the risk of developing left ventricular hypertrophy in patients with treatment-naïve acromegaly.
